# Terahertz detection with an antenna-coupled highly-doped silicon quantum dot

**DOI:** 10.1038/s41598-019-54130-0

**Published:** 2019-12-09

**Authors:** Takuya Okamoto, Naoki Fujimura, Luca Crespi, Tetsuo Kodera, Yukio Kawano

**Affiliations:** 1 Department of Electrical and Electronic Engineering, Tokyo Institute of Technology, 2-12-1 Ookayama, Meguro-ku, Tokyo 152-8552 Japan; 2 Laboratory for Future Interdisciplinary Research of Science and Technology, Tokyo Institute of Technology, 2-12-1, Ookayama, Meguro-ku, Tokyo 152-8552 Japan

**Keywords:** Sensors, Quantum dots

## Abstract

Nanostructured dopant-based silicon (Si) transistors are promising candidates for high-performance photodetectors and quantum information devices. For highly doped Si with donor bands, the energy depth of donor levels and the energy required for tunneling processes between donor levels are typically on the order of millielectron volts, corresponding to terahertz (THz) photon energy. Owing to these properties, highly doped Si quantum dots (QDs) are highly attractive as THz photoconductive detectors. Here, we demonstrate THz detection with a lithographically defined and highly phosphorus-doped Si QD. We integrate a 40 nm-diameter QD with a micrometer-scale broadband logarithmic spiral antenna for the detection of THz photocurrent in a wide frequency range from 0.58 to 3.11 THz. Furthermore, we confirm that the detection sensitivity is enhanced by a factor of ~880 compared to a QD detector without an antenna. These results demonstrate the ability of a highly doped-Si QD coupled with an antenna to detect broadband THz waves. By optimizing the dopant distribution and levels, further performance improvements are feasible.

## Introduction

Doping technologies for semiconductors have a great potential for realizing new functional and high-performance photonic devices. Among the various properties created by dopants, robust quantum states formed by donor levels is one of the most interesting and important. One notable example involves nitrogen-vacancy centers in diamond, which can lead to highly sensitive magnetic sensors and quantum information devices^1^. The use of dopant-assisted photoresponse in silicon (Si) nanodevices is emerging as a promising method for highly sensitive photodetection. Trapping and detrapping of electrons localized in the Si donor levels provides a polarization field, which strongly modifies the local electrostatic environment of percolation pathways in devices. These processes lead to the generation of observable currents, even under very weak light irradiation. By utilizing dopant-based Si nanotransistors, Tabe *et al*. observed single-electron transport through a single dopant^2^ and demonstrated highly sensitive photon detection in the visible light region^3^. In addition, microwave-induced currents have been observed through a doped Si QD channel^4,5^.

Dopant-based Si nanotransistors are opening up new possibilities for high-performance Si photonic devices in the terahertz (THz) regime. For highly doped Si with donor bands, the typical energy depths of individual donor levels are known to be of the order of millielectron volts (meV)^2,4^, which correspond to THz photon energies. Additionally, the neighboring tunneling processes between donor levels also correspond to similar energies^4^. These features provide THz-induced changes in percolation pathways in Si devices due to the modification of the electrostatic environment, leading to the generation of an observable photocurrent^4,5^. Furthermore, this phenomenon occurs because the current in nanostructures is dominated by a smaller number of percolation pathways compared to conventional microscale transistors. Therefore, a highly doped Si QD is appropriate for developing a THz photoconductive detector.

Technologies based on THz waves are becoming increasingly important because of their broad range of potential applications such as security screening, medical checks, and materials characterization^6–8^. Although THz generation and detection based on femtosecond pulse lasers are widely used, solid-state THz devices without pulse lasers are preferable and in demand for practical applications. At present, solid-state THz sources include quantum cascade lasers^9^, resonant tunneling-diode oscillators^10,11^, p-type germanium lasers^12,13^, and superconducting coherent emitters^14,15^. Recently, various nanostructured devices have been employed to enhance THz detector performance. To date, THz detectors with nanoscale devices based on compound semiconductors^16^, superconductors^17,18^, carbon nanotubes^19–21^, and graphene^22–24^ have been reported.

Si-based detectors are one of most reliable devices because they can be entirely fabricated using mature complementary metal-oxide semiconductor-compatible processes. In this paper, we report THz detection based on a lithographically defined and highly phosphorus-doped 40 nm diameter Si QD. One crucial issue concerning the application of such a nanoscale THz detector is that the nanoscale sensing area of a QD is much smaller than the wavelength of THz waves, generally resulting in low THz coupling efficiency. To overcome this problem, we directly integrated a Si QD with a micrometer-scale logarithmic-spiral (log-spiral) antenna^25,26^. We experimentally demonstrated that THz irradiation onto a dopant-based Si QD with a log-spiral antenna generated THz photocurrent in a wide frequency range from 0.58 to 3.11 THz. Furthermore, the detection sensitivity was enhanced by a factor of ~880 compared to a Si QD detector without an antenna.

## Results and Discussion

**Figure 1:**
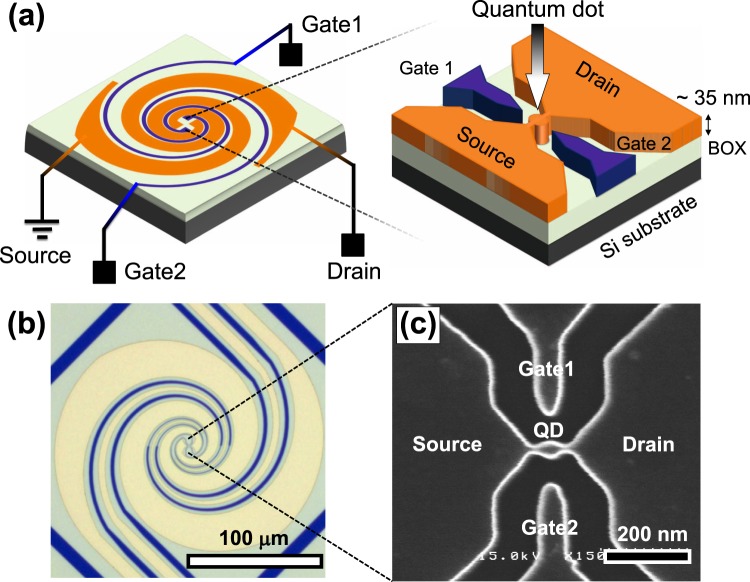
Structure of the THz detector based on the antenna-coupled doped Si quantum dot. (**a**) Schematic of the device structure. (**b**) Optical image of an actual device. (**c**) Scanning electron microscopy image of the Si quantum dot located in the center.

Our highly doped Si QD is depicted in Fig. 1(a), and the fabrication process is described in the Methods section. To efficiently concentrate THz waves on this 40-nm-diameter QD, we integrated a log-spiral antenna, which is a self-complementary broadband antenna^25,26^. An optical image of the device is shown in Fig. 1(b). Furthermore, we employed bowtie-shaped probes in the center of the log-spiral antenna that were connected to source and drain electrodes of the QD, as shown in the scanning electron microscopy image of the QD (Fig. 1(c)). The antenna was made of a Au (100 nm)/Cr (10 nm) film using an electron beam evaporator.

**Figure 2:**
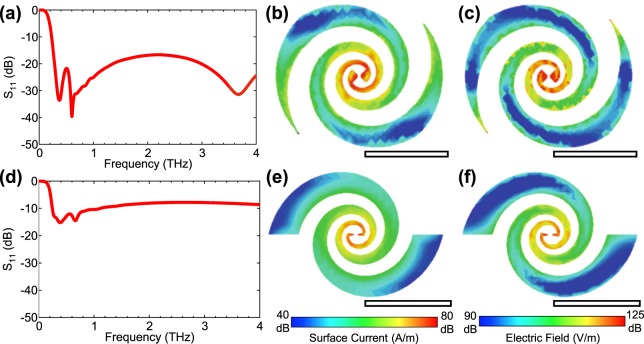
Numerical simulation of the log-spiral antenna with terminated arms (**a–c**) and with non-terminated arms (**d–f**) using a finite integration method. (**a,d**) *S*_11_ parameter versus frequency. (**b,e**) Distribution of surface current at 0.5 THz. (**c,f**) Distribution of electric field at 0.5 THz. The scale bars indicate 100 μm.

Figure 2(a) shows the *S*_11_ parameter of this antenna simulated using a finite integration technique (CST STUDIO) as a function of frequency. The result confirmed that the *S*_11_ parameter exhibited a smooth dependence in a wide frequency range. As shown in Fig. 2(b), the surface current density at 0.5 THz increased toward the center of the antenna because of the reduction in arm size. As a result, the electric fields on the antenna surface were strongly concentrated on the tips of the bowtie-shaped probe. These results indicate that the present structure can work as an antenna for wideband, high-efficiency THz detection with a nanoscale QD photodetector. Recently, this advantage was experimentally demonstrated with scattering-type scanning near-field optical microscopy in the mid-infrared region^27^.

Ideally, a log-spiral antenna has infinitely extended structures^25^. Therefore, to investigate the impact of the arm termination, we performed similar simulations on the non-terminated antenna and plotted the results in Fig. 2(d). The *S*_11_ parameter increased significantly compared to the arm-terminated antenna. As shown in Fig. 2(d), the surface current distribution was similar to that of the arm-terminated antenna in the center region of the antenna. However, at the arm’s end, the current dropped abruptly as shown in Fig. 2(e), leading to reflections. In contrast, the arm termination allowed the current to decrease smoothly along the arms without abrupt drops, thereby reducing reflections and disturbances. Based on the above simulation results, we employed the arm-terminated log-spiral antenna depicted in Fig. 1(a).

**Figure 3:**
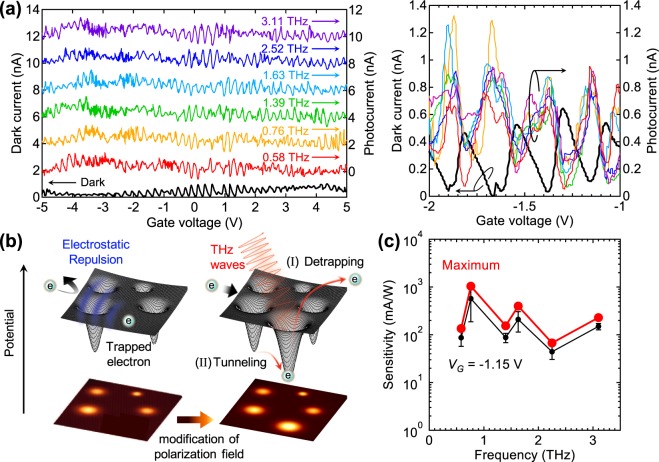
THz response of the highly doped-Si QD with the antenna at six frequencies. (**a**) Dark current *I*_0_ (the dark curve) and THz photocurrent *I*_THz_(*f*) (the other colored curves) at source–drain voltage of 5 mV. THz photocurrent depicted in left figure are offset by multiples of 2 nA. The right figure is smaller *V*_*G*_ range. (**b**) Schematic view of the THz responses in the Coulomb blockade region; (**I**) detrapping process of the localized electron in donor levels; (II) THz-assisted tunneling process between the donor levels. These processes provide the modification of polarization field as shown in the bottom plains. (**c**) Frequency dependence of the maximum and *V*_*G*_-fixed sensitivity.

We measured the DC currents passing through the QD at a source–drain voltage of 5 mV and THz-induced photocurrent *I*_THz_(*f*) = *I*_all_(*f*) − *I*_0_, where *I*_0_ is the dark current without THz illumination and *I*_all_(*f*) is the total current with THz illumination at a frequency of *f*. All measurements were performed at ~3 K (corresponding to 0.26 meV and 0.063 THz), as described in the Methods section. Figure 3(a) shows the dark current and the THz-induced photocurrent as functions of the gate voltage *V*_*G*_. Periodic dark current oscillations relative to *V*_*G*_ (Coulomb oscillations) were observed, indicating that the QD operated as a single-electron transistor.

Interestingly, the THz photocurrent was mainly observed in the Coulomb blockade regions where electron tunneling was blocked as shown in the right panel of Fig. 3(a). Since its peak position relative to *V*_*G*_ does not depend on the frequency of the illuminated THz waves, the THz photocurrent was not mainly caused by photon-assisted tunneling; this is due to the quantum level in zero-dimensional materials^20,21,28^. Here, we propose two mechanisms due to high doping for photocurrent generation in our device: (1) Detrapping processes from individual localized energy states created by the donor levels depicted as (I) in Fig. 3(b). This typical energy depth is known to be of the order of meV^2,4^, which corresponds to THz photon energy. Note that this energy depth effectively decreases compared to that of isolated donor (~45 meV of phosphorus), because the donor level broadens into bands^29^. (2) THz-assisted tunneling processes to empty donor levels^4^ depicted as (II) in Fig. 3(b). Both of these processes lead to a THz-induced polarization field and strongly modifies the local electrostatic environment of percolation pathways in the device, leading to the generation of observable photocurrent as theoretically shown in the Supplementary Information. Due to the confined electronic system of the QD with few percolation pathways, these pathways can be strongly modified further.

Figure 3(c) plots the maximum and *V*_*G*_-fixed detection sensitivity (i.e., THz photocurrent divided by the THz power onto the device) as a function of frequency. The *V*_*G*_-fixed sensitivity was determined by Lorentzian fitting at *V*_*G*_ = −1.15 V. A smooth frequency dependence throughout the measured frequency range is clearly observed. This result confirms that the highly doped QD integrated with a log-spiral antenna can detect THz waves in a broad frequency region. This broadband response is attributed to both the broadband property of the antenna and the donor bands with the finite energy widths in the Si. THz detection in different or wider frequency regions are possible by adjusting the antenna parameters associated with cutoff frequencies. Considering the noise current density of this device (~10^−13^ A/Hz^1/2^), we roughly estimated that the noise equivalent power (NEP) is ~10^−13^ W/Hz^1/2^. We presume that the NEP is potentially lower, because the noise from the measuring system (e.g. the mechanical refrigerator) largely contributed to the noise value measured in this work.

**Figure 4:**
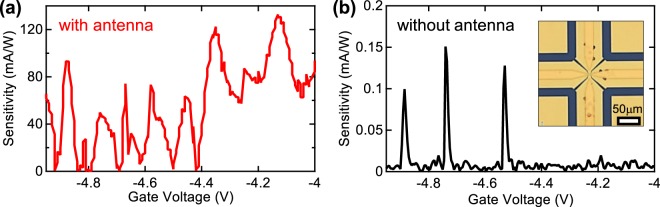
Detection sensitivity at 3.11 THz of the antenna-coupled QD (**a**) and the non-antenna-coupled QD (**b**). The inset in (**b**) shows an optical image of the non-antenna-coupled device.

In order to verify the effect of the antenna, we made similar measurements of a QD device without an antenna. The non-antenna QD device was fabricated using the same processes used for the antenna-coupled QD device. Figure 4 compare the detection sensitivity between the two types of QD detectors at 3.11 THz. In Fig. 4, the maximum detection sensitivity of the antenna-coupled QD was 132.7 mA/W, whereas that of the non-antenna QD was 0.15 mA/W. Thus, the enhancement factor resulting from the presence of the log-spiral antenna was approximately 880. These results verify that the antenna efficiently focuses THz waves on the nanoscale QD photodetector.

In conclusion, we have demonstrated THz wideband detection using a highly doped Si QD device with a log-spiral antenna. The highly doped Si QD’s broadband capability is a result of both the dopant level and the log-spiral antenna’s broadband properties. It may possible to have an even more sensitive THz using the following two methods. One is by improving the read-out efficiency through the implementation of single-charge sensing with another single-electron transistor integrated in the proximity of the QD detector^30^ to enable the detection of THz photocurrent on the single-electron level. The other solution is by improving the quantum efficiency by sophisticated Si doping techniques such as deterministic doping^31^ to control the donor bands’ energy spread. In addition, by using a variety of donors and acceptors, it is possible to operate in a different bandwidth such as in the near-infrared range by using a deep level dopant (e.g., ~0.51 eV Ge-vacancy center as a donor^32^). An additional advantage of using Si nanotechnology is that arrayed Si-QD detectors are compatible with mature Si integrated circuit technologies for multiple signal processing and amplification techniques, which can then be applied to THz cameras in the future.

## Methods

### Sample fabrication

The THz detector was fabricated on a silicon-on-insulator (SOI) substrate. The QD structure and the log-spiral antenna were patterned using lithography. As illustrated in Fig. 1(a), the thickness of the Si layer in the SOI substrate was reduced to approximately 35 nm by thermal oxidation, contributing to the increase in the charging energy of the QD^30^. The QD, source, drain, and gate parts were doped via phosphine gas flow. The dopant concentration was 1 × 10^19^ cm^−3^.

### Measurements

The temperature of the Si-QD THz detector was decreased to approximately 3 K using a mechanical refrigerator (NIKI GLASS Co., LTD). The electrical measurements were conducted using a voltage source (Hewlett-Packard Company, 3245 A), a voltage meter (Agilent Technologies International Japan, Ltd, 34401 A), and current amplifier. (DL Instruments, LLC, Model 1211). For measuring the THz photocurrent of the non-antenna-coupled device, the THz signal was further amplified by a lock-in amplifier (NF Corporation, LI5640) due to the very low signal, where the THz waves were chopped at 40 Hz.

We used a molecular gas laser pumped by a CO_2_ mid-infrared laser as a THz illumination source. The frequency of the THz source could be varied by either tuning the wavelength of the CO_2_ pump laser or changing the type of gas. In this experiment, six frequencies (0.58, 0.76, 1.39, 1.63, 2.52, and 3.11 THz) were used. The beam was focused and guided by an off-axis parabolic mirror through a silver-coated pipe (3 mm core and 1.2 m) and Tsurupica window (7 mm thickness) at room temperature and filtered by a black-polyethylene film at low temperature. The transmission factors were estimated to be ~0.7 for the silver-coated pipe, ~0.6 for the Tsurupica window, and 0.4 for the black-polyethylene film using a power meter (Ophir Optronics, NOVA II). The THz power onto the devices was derived to be several tens of nW, where the effective photoactive area was approximated as a square of the THz wavelength.

The sensitivity was derived by dividing the THz photocurrent by the THz power onto the detector. The NEP was calculated to be noise current density (in A/Hz^1/2^)/sensitivity (in A/W), where the noise current density was estimated in the Coulomb blockade region.

## Supplementary information

Supplementary Information
