# Supplementary material for: Terahertz detection with an antenna-coupled highly-doped silicon quantum dot
Source: Sci Rep. 2019 Dec 9;9:18574. doi: 10.1038/s41598-019-54130-0 (PMC6901460; doi:10.1038/s41598-019-54130-0)
Supplement: Supplementary file 1 — Supplementary Information [file 41598_2019_54130_MOESM1_ESM.pdf]

Supplementary information: Terahertz detection with an antenna-coupled highly-doped silicon quantum dot

Takuya Okamoto[1,2], Naoki Fujimura[1,2], Luca Crespi[1,2], Tetsuo Kodera[1], and Yukio Kawano[1,2,*]

[1]Department of Electrical and Electronic Engineering, Tokyo Institute of Technology, 2-12-1 Ookayama, Meguro-ku, Tokyo 152-8552, Japan
[2]Laboratory for Future Interdisciplinary Research of Science and Technology, Tokyo Institute of Technology, 2-12-1, Ookayama, Meguro-ku, Tokyo 152-8552, Japan
[*]kawano@pe.titech.ac.jp

THEORETICAL MODEL

We theoretically discuss the system of our device; especially we show the reason why the photocurrent appears under the THz waves (Fig. 3(b)). This system consists of the two-level system (TLS) with trapped $|1\rangle$ and de-trapped $|2\rangle$ states, and external THz waves (AC external electric field with THz frequency), which can be expresses by the following Hamiltonian $\hat{H}$

$$\hat{H} = \hat{H}_{\mathrm{TLS}} + \hat{H}_{\mathrm{ext}} \tag{1}$$

where $\hat{H}_{\mathrm{TLS}}$ and $\hat{H}_{\mathrm{ext}}$ represent the Hamiltonian of the TLS and of its interaction with the external THz waves with the frequency ω_{ext}, respectively. The wavefunction of this system is described as $|\Psi(t)\rangle = c_1|1\rangle + c_2 \mathrm{e}^{-i\omega_{\mathrm{ext}}t}|2\rangle$. The population of each state is described using density matrix $\rho(t) = |\Psi(t)\rangle\langle\Psi(t)|$, whose elements are $\rho_{mn} = c_m c_n^*(m,n=1,2)$.

The first term of Eq. (1) is given by $\hat{H}_{\mathrm{TLS}} = (\hbar\omega_{12}/2)\sigma_z$, where $\hbar$ is Planck constant divided by 2π, ω_{12} is transition frequency of the TLS, and $\sigma_z = \mathrm{diag}(1,-1)$ is Pauli matrix. The secound term of Eq. (1) is expressed as $\hat{H}_{\mathrm{ext}} = -\boldsymbol{\mu}\cdot\boldsymbol{E}_{\mathrm{ext}}(t) = -\boldsymbol{\mu}\cdot\boldsymbol{E}_0\mathrm{e}^{i\omega_{\mathrm{ext}}t}$ using dipole moment $\boldsymbol{\mu}$; where we used dipole approximation because this system is much smaller than wavelength of the THz waves. To describe $\hat{H}$ with the interaction picture as $\hat{H}_i$, we performed a unitary transformation $\hat{U} = \exp(-i\hat{H}_{\mathrm{TLS}}t/\hbar)$;

$$\hat{H}_i = \hat{U}^\dagger(\hat{H} - \hat{H}_{\mathrm{TLS}})\hat{U} \tag{2}$$

$$= -\Delta|1\rangle\langle 1| - \hat{U}^\dagger(\boldsymbol{\mu}\cdot\boldsymbol{E})\hat{U}\mathrm{e}^{i\omega_{\mathrm{ext}}t} \tag{3}$$

$$= \frac{1}{2}\begin{pmatrix} 0 & -\langle 1|\boldsymbol{\mu}|2\rangle\cdot\boldsymbol{E}_0(1+\mathrm{e}^{-2i\omega_{\mathrm{ext}}t}) \\ -\langle 2|\boldsymbol{\mu}|1\rangle\cdot\boldsymbol{E}_0(1+\mathrm{e}^{2i\omega_{\mathrm{ext}}t}) & -2\hbar\Delta \end{pmatrix} \tag{4}$$

where $\Delta = \omega_{\mathrm{ext}} - \omega_{12}$ is the detuning between the transition frequency of the TLS and of the external THz waves. By using rotating-wave approximation, $\hat{H}_i$ is deformed into $\hat{H}_{\mathrm{RWA}}$ without time dependence

$$\hat{H}_{\mathrm{RWA}} = \frac{1}{2}\begin{pmatrix} \hbar\Delta & -\langle 1|\boldsymbol{\mu}|2\rangle\cdot\boldsymbol{E}_0 \\ -\langle 2|\boldsymbol{\mu}|1\rangle\cdot\boldsymbol{E}_0 & -\hbar\Delta \end{pmatrix} \tag{5}$$

$$= -\frac{\hbar}{2}\begin{pmatrix} -\Delta & \Omega \\ \Omega & \Delta \end{pmatrix} \tag{6}$$

where $\Omega = \langle 1|\boldsymbol{\mu}|2\rangle\cdot\boldsymbol{E}_0/\hbar$ is the Rabi rate, which is a positive real value owing to adequate adjustment of the phase of the wavefunction.

In the TLS, damping processes have to be teken into account. To describe such processes phenomenologically, the motion equation of $\rho(t)$ is expressed with the $\hat{H}$:

$$\partial_t\rho(t) = -\frac{i}{\hbar}\left[\hat{H}_{\mathrm{TLS}} + \hat{H}_{\mathrm{RWA}}, \rho(t)\right] \tag{7}$$

Here, the two damping processes are conceivable: One is the THz-induced emission and absorption processes with rates Γ, and another is intrinsic dephasing process with rates γ. These damping processes are phenomenologically introduced as:

$\gamma_a = \Gamma/2 + \gamma$. By using these damping rate, Eq. (7) is transformed as

$$\partial_t \rho(t) = \begin{pmatrix} -i\frac{\Omega}{2}(\rho_{21}-\rho_{12}) + \Gamma\rho_{22} & -i\frac{\Omega}{2}(\rho_{22}-\rho_{11}) - (\gamma_a + i\Delta)\rho_{12} \\ i\frac{\Omega}{2}(\rho_{22}-\rho_{11}) - (\gamma_a - i\Delta)\rho_{21} & i\frac{\Omega}{2}(\rho_{21}-\rho_{12}) - \Gamma\rho_{22} \end{pmatrix} \tag{8}$$

To discuss the occurence of the THz-induced current in the following section, the steady-state solution is solved by setting the time derivatives to 0.

$$\rho_{21}^{\mathrm{ste}} = \frac{\Omega}{2} \frac{\Gamma(\Delta - i\gamma_\alpha)}{\Gamma(\gamma_\alpha^2 + \Delta^2) + \gamma_\alpha \Omega^2} \tag{9}$$

Here, let us discuss the THz-induced photocurrent $I_{\mathrm{THz}}(t)$. Since in the intrinsic dephasing a typical dopant energy level corresponds to a THz photon energy, we assume that complex conductivity $\sigma(t)$ is expressed as $\sigma(t) = \sigma_0 \exp\{(-i\omega_b - \Gamma_b)t\}$, where $I_{\mathrm{THz}}(t)$ is resonant with the frequency ω_b and dephasing rate Γ_b. Through linear response theory, $I_{\mathrm{THz}}(t)$ is given by:

$$I_{\mathrm{THz}}(t) = \int_0^t \sigma(t-t')E(t')\mathrm{d}t' \tag{10}$$

Through the time-derivative of Eq. (10), the motion equation of $I_{\mathrm{THz}}(t)$ can be obtained as:

$$\partial_t I_{\mathrm{THz}}(t) = -(i\omega_b + \Gamma_b)I_{\mathrm{THz}}(t) + \sigma_0 E(t) \tag{11}$$

$E(t)$ is the total electric field having two contributions: one is the external THz field $E_{\mathrm{ext}}(t) = E_0 \exp(i\omega_{\mathrm{ext}}t)$, and another is the field induced by the ionized dopants with polarization $P(t)$:

$$P(t) = \langle \Psi(t) | -\boldsymbol{\mu} | \Psi(t) \rangle \tag{12}$$

$$= \mu \rho_{21} \mathrm{e}^{i\omega_{\mathrm{ext}}t} \tag{13}$$

$P(t)$ effectively contains photon-assisted tunneling between donor levels, because this process requires the empty donor levels created by de-trapping processes through Pauli exclusion principle. Substituting Eq. (13) into Eq. (11) yields

$$\partial_t I_{\mathrm{THz}}(t) = -(i\omega_b + \Gamma_b)I_{\mathrm{THz}}(t) + \sigma_0 (E_0 + \mu\rho_{21}/\varepsilon_r) \mathrm{e}^{i\omega_{\mathrm{ext}}t} \tag{14}$$

where ε_r represents the dielectric function of highly doped Si. Introducing $\tilde{I}_{\mathrm{THz}}(t) = I_{\mathrm{THz}}(t)\mathrm{e}^{-i\omega_{\mathrm{ext}}t}$ removes the fast rotating term $\mathrm{e}^{i\omega_{\mathrm{ext}}t}$; which transform the Eq. (14) to

$$\partial_t \tilde{I}_{\mathrm{THz}}(t) = i(\Delta + \delta)\tilde{I}_{\mathrm{THz}}(t) - \Gamma_b \tilde{I}_{\mathrm{THz}}(t) + \sigma_0 (E_0 + \mu\rho_{21}/\varepsilon_r) \tag{15}$$

where, $\delta = \omega_{12} - \omega_b$ is the frequency difference between the TLS and the background resonance. The steady current I^{ste} can be derived by setting the time derivatives to 0 in Eq. (15) as:

$$I_{\mathrm{THz}}^{\mathrm{ste}} = \frac{\sigma_0}{\Gamma_b - i(\Delta + \delta)} (E_0 + \mu\rho_{21}^{\mathrm{ste}}/\varepsilon_r) \tag{16}$$

This result of Eq. (16) gives an analytical description of THz-induced photocurrent.

In the Coulomb blockade regions, THz-induced photocurrent is expressed by Eq. (16) with the second term of transition processes ρ_{21}^{ste} governed by Eq. (9). Meanwhile, in the resonant transmission regions where the current peak is observed, the eigenstates are almost governed by de-trapped states $|2\rangle$. Since this situation leads to the strong suppression of transition processes $\rho_{21} \sim 0$, the second term in the Eq. (16) is negligible. The above-mentioned discussion suggests that the Fermi-level dependence of ρ_{21} yields the gate-voltage dependence of the THz-induced photocurrent, thus indicating that the THz-induced photocurrent observed in the Coulomb blockade regions mainly originates from the transition processes in the TLS of the trap/de-trapped states.

Equation (16) also explains the difference of THz-induced photocurrent between the antenna-coupled and non-antenna QD devices. Since the QD device without such an antenna can not couple with the THz waves efficiently, the THz electric field on the non-antenna QD device is much weaker than that on the antenna-coupled QD device. This fact directly results in the decrease in the contribution of the first term in Eq. (16). Additionally, such weak electric field hardly excites the electrons trapped in individual dopant potentials; hence the eigenstates are almost determined by trapped states $|1\rangle$ and consequently ρ_{21} becomes very small. For this reason, the THz-induced photocurrent of Eq. (16) without the antenna is much smaller than that with the antenna.
